# Aging exacerbates neutrophil pathogenicity in ischemic stroke

**DOI:** 10.18632/aging.102632

**Published:** 2020-01-12

**Authors:** Meaghan A. Roy-O’Reilly, Hilda Ahnstedt, Monica S. Spychala, Yashasvee Munshi, Jaroslaw Aronowski, Lauren H. Sansing, Louise D. McCullough

**Affiliations:** 1 Department of Neurology, University of Texas Health Science Center, Houston, TX 77030, USA; 2 Department of Neurology and Center for Neuroepidemiology and Clinical Neurological Research, Yale School of Medicine, New Haven, CT 06520, USA

**Keywords:** neuroinflammation, aging, ischemic stroke, ischemia, immunology

## Abstract

Ischemic stroke is major cause of disability and mortality worldwide, and aging is strong risk factor for poor post-stroke outcome. Neutrophils traffic rapidly to the brain following ischemic stroke, and recent evidence has suggested that aging may alter neutrophil function after tissue injury. In this study, we hypothesize that aging enhances the pro-inflammatory function of neutrophils, directly contributing to the poorer outcomes seen in aging patients. We utilized demographic data and biological specimens from ischemic stroke patients and an experimental mouse model to determine the correlation between age, neutrophil function and stroke outcomes. In ischemic stroke patients, age was associated with increased mortality and morbidity and higher levels of neutrophil-activating cytokines. In mice, aged animals had higher stroke mortality and morbidity, higher levels of neutrophil-activating cytokines and enhanced generation of neutrophil reactive oxygen species compared to young mice. Finally, depletion of neutrophils via a specific monoclonal antibody after ischemic stroke led to long-term benefits in functional outcome in aged male and female animals, with no benefit observed in young. These results demonstrate that aging is associated with augmented neutrophil pathogenicity in ischemic stroke, and that neutrophil-targeted therapies may confer greater benefit in aged subjects.

## INTRODUCTION

Stroke is the 2^nd^ leading cause of death worldwide [1]. Acute ischemic stroke, accounting for ~87% of all strokes, results from the loss of cerebral blood flow [2]. Importantly, secondary damage pathways can exacerbate tissue injury for days or weeks after the initial insult [3]. A growing body of evidence suggests that sterile inflammation plays a major role in secondary damage after ischemic stroke [4]. Of particular interest are neutrophils, innate immune cells that are released from the bone marrow and recruited to the brain following ischemic stroke [5]. During conditions of infection, neutrophils have been shown to protect host tissues via phagocytosis, degranulation, neutrophil extracellular trap release and reactive species generation [6]. However, in conditions of excessive inflammation such as ischemic stroke, these neutrophil defenses are believed to exacerbate tissue damage. [7].

Numerous pre-clinical studies have shown that blocking neutrophil-associated processes like reactive species production can reduce infarct volume after stroke, yet clinical trials targeting neutrophils have failed to show benefit in ischemic stroke patients [5]. The widespread use of young animals in pre-clinical studies has been identified as a potential cause of poor clinical translation, as ischemic stroke is largely a disease of aging [8, 9]. Aging is associated with altered stroke pathology, worse functional outcomes and diminished recovery in both human stroke patients and experimental models [10–12]. Evidence suggests that the use of aged animals in pre-clinical stroke studies may better replicate pathophysiology in patients [13].

We believe that age represents a critical variable in the study of neutrophils in stroke, as aging itself can alter neutrophil function [14–16]. Our lab has previously shown that aged brain-infiltrating neutrophils have heightened pro-inflammatory functions after stroke, including elevated reactive species production [17]. However, the mechanism and impact of age-related changes in neutrophil function on ischemic stroke outcome remains unknown.

In this study, we hypothesized that aging enhances the pathogenicity of neutrophils in ischemic stroke, contributing to poorer outcomes in older subjects. We utilized the MCAO mouse model of ischemic stroke in young and aged mice, in addition to a large stroke patient cohort, to determine whether age influenced neutrophil quantity or inflammatory phenotype after stroke. Next, we used flow cytometry to explore whether age altered neutrophil reactive species production in a cell-intrinsic or environment-dependent manner. In addition, we examined whether age altered the levels of neutrophil-activating cytokines in the circulation after experimental ischemic stroke, followed by confirmatory measurements in ischemic stroke patients. Finally, we used a neutrophil-specific depleting antibody to test the contribution of neutrophils to ischemic stroke pathology in both young and aged mice.

## RESULTS

### Age influences stroke incidence, mortality and morbidity in human patients

**Figure 1:**
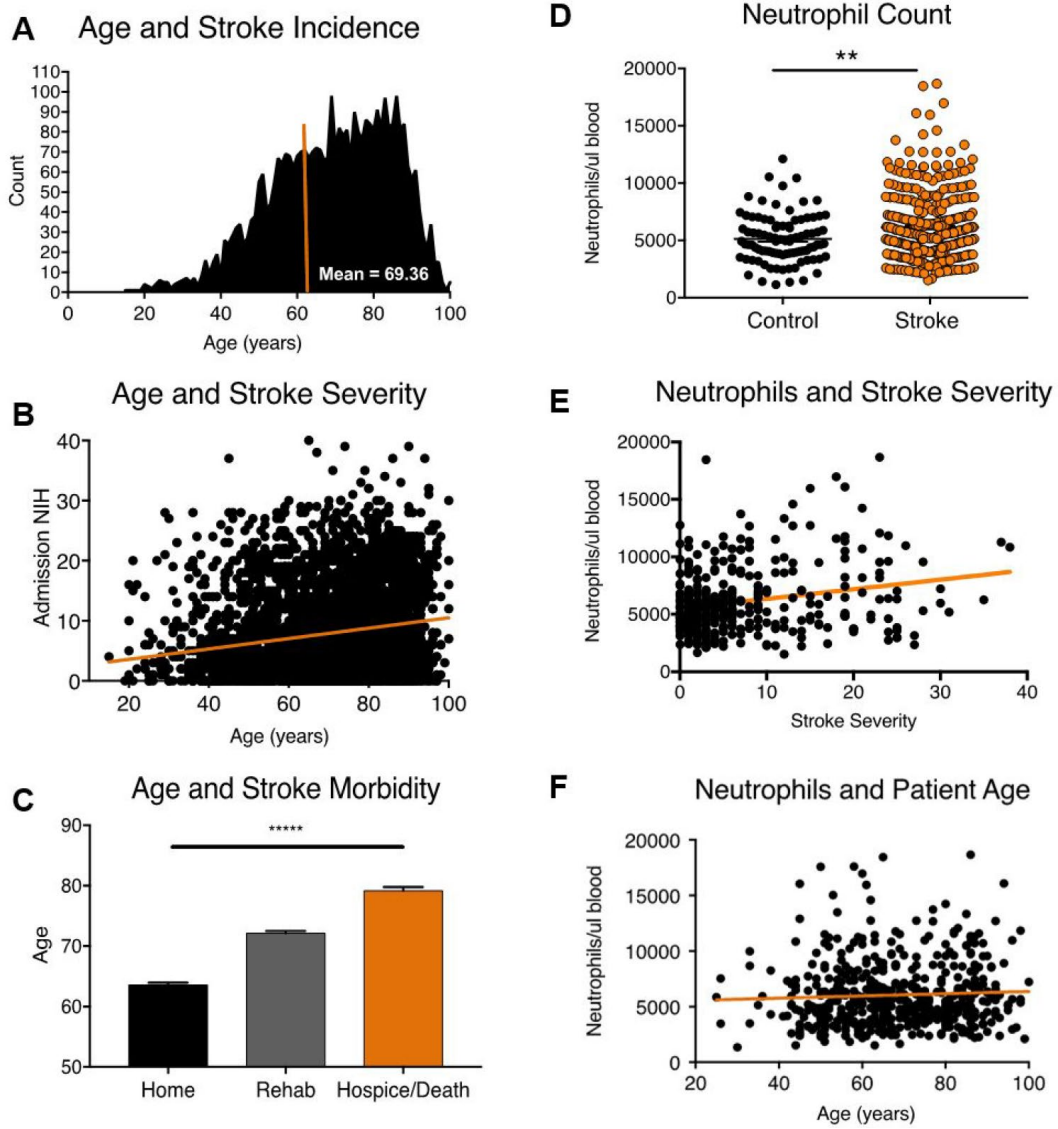
**Age, stroke incidence, stroke outcome and 24-hour neutrophil counts in patients admitted to a major stroke center.** (**A**) Distribution of age at stroke onset (n=3635). (**B**) Correlation of age vs. baseline stroke severity as measured by admission NIH stroke scale (n=3635). (**C**) Relationship between patient age and stroke outcome at discharge (n=3635). (**D**) Mature neutrophil counts in stroke (n=508) and TIA control patients (n=130). (**E**) Mature neutrophil count and stroke severity, as measured by NIHSS on admission (R^2^ =0.04, n=508, p= p<0.0001). (**F**) Mature neutrophil counts and stroke patient age (R^2^ =0.002, n=508, p=.2). *p≤0.05, **p=<0.01, ***p=<0.001, ****p=<0.0001.

To study the association of age with mortality and morbidity after acute ischemic stroke, we conducted a retrospective chart review of patients admitted to a primary stroke care center over a 10-year period, utilizing inclusion and exclusion criteria as described in the methods section (n= 3635, Figure 1, Supplementary Table 1). The average age of stroke patients in our demographic cohort was 69 years (Figure 1A). Increasing age was significantly associated with greater initial stroke severity (Figure 1B, F value 115.7, DFn, d 1,3633, linear regression, p<0.0001), as measured by the NIH Stroke Scale. Using discharge disposition as a measure of functional outcome, we found that patients with low morbidity (discharge home) were significantly younger (63.57 ± 15.01 years) compared to patients in the high morbidity (discharge to rehab, 72.15 ± 14.75 years) and mortality (hospice or death, 79.2 ± 12.56 years) groups (Kruskall-Wallis test, p<0.0001, Figure 1C), consistent with global statistics [2].

### Age alters circulating neutrophil quality, but not quantity, after acute ischemic stroke

As age may alter (1) neutrophil numbers or (2) neutrophil function in ischemic stroke, we then examined whether age was associated with acute post-stroke neutrophilia in patients. Ischemic stroke patients from the demographic cohort with available neutrophil counts within 24 hours of symptom onset were selected for this analysis (n= 508), with transient ischemic attack (TIA) patients serving as controls (n=130). As predicted, neutrophil counts were significantly higher in ischemic stroke patients (6049 ± 2952 neutrophils/ul) compared to TIA controls (4955 ± 1984 neutrophils/ul) 24 hours after symptom onset (Mann-Whitney test, p=0.0005, Figure 1D). Within ischemic stroke patients, neutrophil count was positively correlated with stroke severity (Figure 1E, F value 33.32, DFn, d 1, 506, linear regression, p<0.0001), but was not significantly associated with patient age (Figure 1F, F value 1.355, DFn, d 1, 506, linear regression, p=0.25). Multivariate multiple regression analysis controlling for patient age and sex confirmed the association between neutrophil count and stroke severity (p=<0.0001, Supplementary Table 2), with no influence of patient age on post-stroke neutrophil count seen.

### Age exacerbates poor stroke outcome in a mouse model

Next, we replicated the age-related increase in ischemic stroke mortality and morbidity in an experimental mouse model. Young (3 month) and aged (22 month) mice were subjected to 60-minute middle cerebral artery occlusion (MCAO) or sham surgery with equivalent reductions in cerebral ischemia (Supplementary Table 3), consistent with our previous work [13].

**Figure 2:**
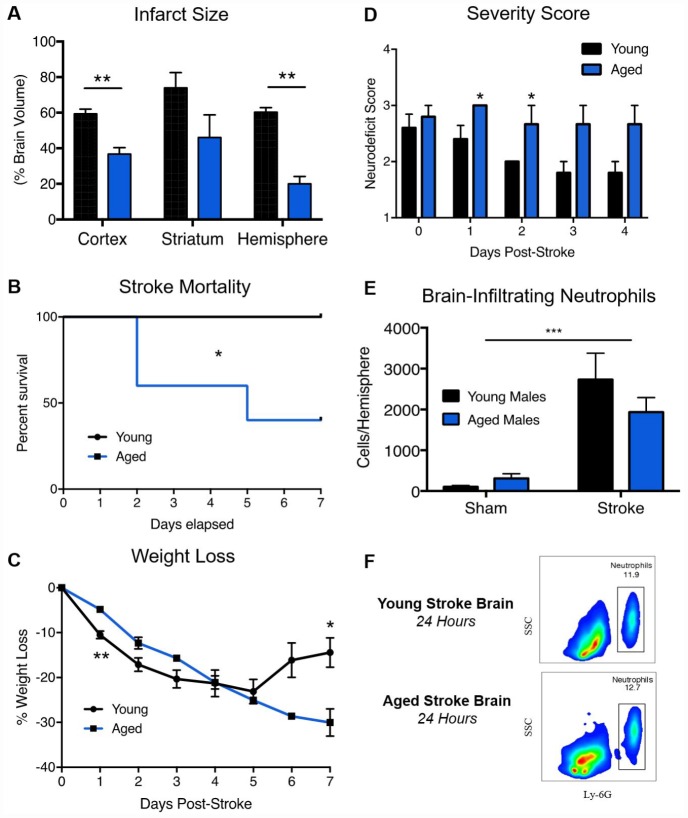
**Aged animals experience smaller infarcts, poorer neurological deficits and equivalent neutrophil infiltration than young animals following ischemic stroke.** Young (3 month) and aged (24 month) mice were subjected to 60-minute MCAO. (**A**) Infarct volumes by TTC 24 hours post-stroke, n=7-11/group, presented as mean +/- SEM. (**B**) Stroke mortality rates, n=5/group, (**C**) Weight loss as a % of starting weight, n=5/group (**D**) Neurodeficit scores, n=5/group. (**E**) Absolute quantification of brain-infiltrating neutrophils in sham and stroke animals 24 hours after stroke onset, (**F**) Representative flow plots. SSC=Side Scatter. n=3/sham group, 4/stroke group. *p≤0.05, **p=<0.01, ***p=<0.001.

Ischemic stroke histology data from young and aged animals is presented in Figure 2. Consistent with prior studies, young animals had significantly larger infarct volumes (Student T-Test, p=.04) at 24 hours post-stroke compared to aged animals (Figure 2A) [13, 17–20]. In a separate cohort used to assess sub-acute outcomes, post-stroke mortality at 7 days was significantly higher in aged animals compared to young (Figure 2B, 60% vs. 0%, df 1, Mantel-Cox test, p=0.05). Although weight loss was initially higher in young animals due to lower starting weight, aged animals exhibited poorer weight recovery at day 7 (Figure 2C, Student T-Test, p=0.04). Neurodeficit scores, a measure of gross neurological dysfunction, were significantly worse in aged animals at day 1 and 2 post-stroke (Figure 2D, Student T-Test, p=0.03). These results are congruent with detailed behavioral testing previously done by our lab, demonstrating overall poorer outcomes for aged animals following ischemic stroke [13].

### Aging does not alter the proportion of neutrophils in circulation after ischemic stroke

To echo our human studies, we next examined whether age influenced neutrophil quantity after ischemic stroke. Although stroke has been shown to exacerbate the release of neutrophils from the bone marrow, [5] no published data has yet examined the effects of aging on neutrophilia after stroke across organ systems. Flow cytometry was conducted in the lungs, bone marrow and spleens from young and aged mice 24 hours after stroke or sham surgery (Supplementary Figure 1). Stroke resulted in an overall increase in neutrophil proportions in the peripheral blood, lungs and spleen, with a significant decrease in neutrophil proportions in the bone marrow after stroke. Aged animals had higher neutrophil proportions in the bone marrow and lung than young animals under both sham and stroke conditions, with a trend towards a similar pattern in the spleen. However, in line with our human stroke patient data, no effect of age was seen on neutrophil proportions in the blood after acute ischemic stroke.

### Age does not increase neutrophil infiltration into the brain 24 hours post-stroke

As changes in the circulation may not accurately reflect neutrophil infiltration into the brain, absolute neutrophil counts in the ischemic brain 24 hours after stroke onset were quantified by flow cytometry (Figure 2E, 2F), with neutrophils identified as CD45^High^/CD11b^+^/Ly6G^+^ cells (gating strategy shown in Supplementary Figure 2). Stroke significantly increased the number of neutrophils in the brain in both age groups, but no significant age difference in neutrophil infiltration into the brain after stroke was observed (Figure 2E, Two-Way ANOVA, Stroke Effect p=0.0007, Age Effect p=0.5164, Interaction p=0.218).

### Age does not increase the production of reactive species in naïve bone marrow neutrophils

**Figure 3:**
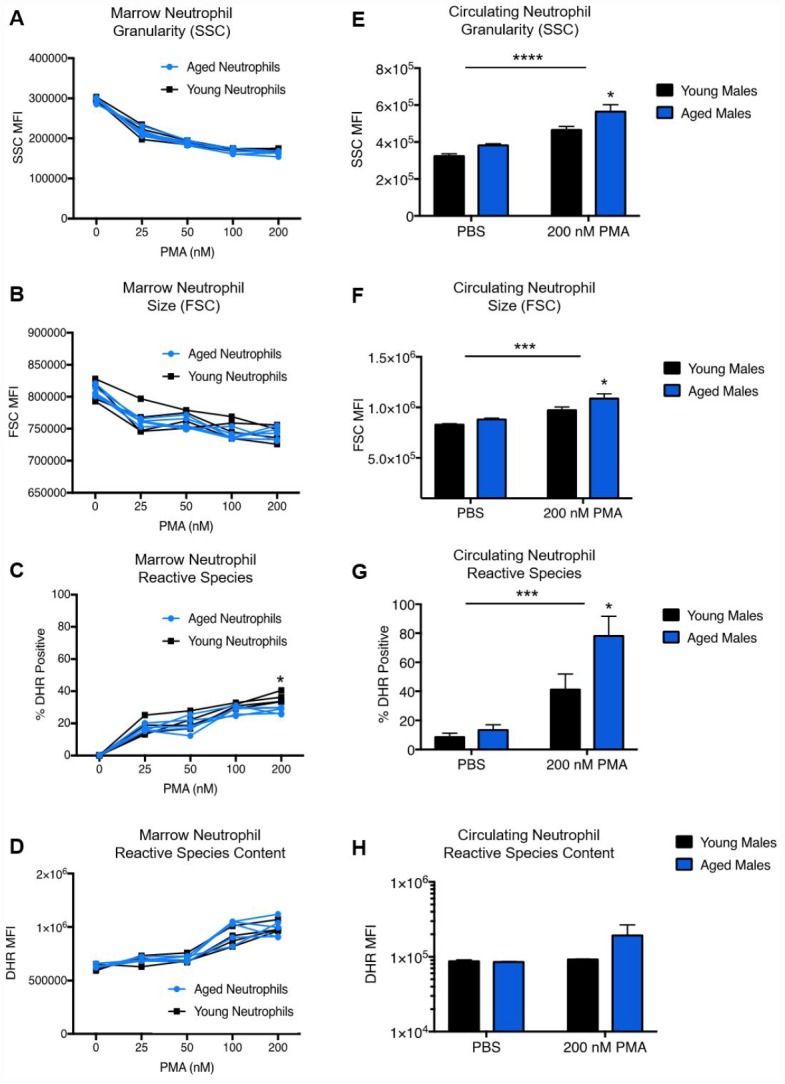
**Age does not affect neutrophil granularity, size or reactive species content in the bone marrow.** Neutrophils from young (3 month) and aged (22-24 month) mice were isolated from the bone marrow of both hind femurs. Cells were exposed to increasing concentrations of PMA for 45 minutes, than analyzed for granularity (**A**), size (**B**), reactive species positivity (**C**) and intracellular reactive species content (**D**). Neutrophils from the blood of naïve young and aged mice were stained for neutrophil identification markers (CD45^+^/CD11b^+^/Ly6C^Int^/Ly6G^Hi^), followed by incubation with or without PMA in the presence of 1,2,3 DHR to measure changes in (**E**) neutrophil granularity (SSC) and (**F**) neutrophil size (FSC), as well as the percentage of ROS positive neutrophils (**G**) and intracellular ROS production (**H**). *p=<.05, **p=<.01, ***p=<0.001, ****p=<0.0001.

Our lab has previously shown that aged brain-infiltrating neutrophils produce higher levels of reactive species than young neutrophils after ischemic stroke. However, it remains unknown whether naïve neutrophils from aged animals inherently possess a higher capacity for reactive species generation. To test this, we isolated neutrophils from the bone marrow of young and aged mice and measured their production of reactive species upon stimulation with phorbol 12-myristate 13-acetate (PMA), a powerful activator (Figure 3). No significant difference in granularity (Figure 3A), size (Figure 3B) or intracellular reactive species content (Figure 3D) was seen between young and aged neutrophils. A slight increase in the % of reactive species positive neutrophils in young animals compared to aged animals was seen after stimulation with 200 nM PMA (Figure 3C, multiple T-Test, corrected p value =0.01). Follow up studies also found no age difference in the production of neutrophil-derived cytokines and chemokines (Supplementary Table 4, Supplementary Figure 3) by multiplex cytokine analysis.

### Age augments neutrophil activation and reactive species production in circulating neutrophils

As we observed no significant age differences in reactive species generation in bone marrow neutrophils, it is possible that age differences in neutrophil function may result from environmental factors rather than cell-intrinsic processes. Aging has been suggested to result in an increase in circulating pro-inflammatory cytokines, a process known as “inflammaging”. To this end, we hypothesized that neutrophils from the bone marrow might begin to exhibit enhanced oxidative functions after exposure to enhanced pro-inflammatory signals in the aged circulation. Circulating murine neutrophils from naïve mice were exposed to 200 nM PMA or PBS control (Figure 3). Stimulation with 200nM PMA resulted in a significant increase in neutrophil side scatter (SSC), a marker of enhanced granularity/activation, in both groups. Interestingly, aged neutrophils were found to have significantly higher SSC than young neutrophils across groups (two-way ANOVA, interaction p=0.3758, stim p=<0.0001, age p=0.0078), which remained significant in the stimulated group after multiple comparisons analysis (p=0.02). A similar pattern was seen in neutrophil forward scatter (FSC) by two-way ANOVA (Figure 3F, interaction p=0.3202, stim p=0.0004, age p=0.0229), with significantly higher FSC in stimulated aged neutrophils compared to young neutrophils after stimulation by multiple comparisons (p=0.05). Measurement of intracellular reactive species production showed that a significantly higher percentage of aged neutrophils were positive for intracellular reactive species after PMA stimulation (Figure 3G, two-way ANOVA with multiple comparisons, p=0.0382), with a trend towards increased reactive species content (Figure 3H, two-way ANOVA with multiple comparisons, p=0.1782) compared to young.

### Age reduces the expression of receptors and ligands required for bone marrow homing and clearance of circulating neutrophils

**Figure 4:**
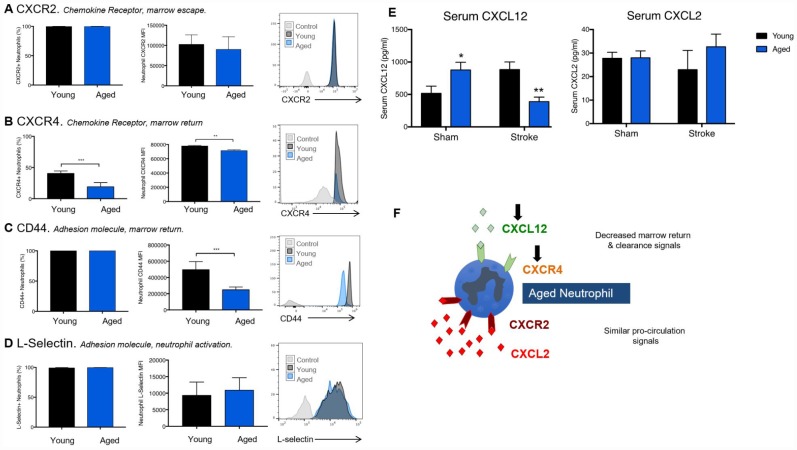
**Age reduces expression of receptors and ligand required for proper neutrophil clearance.** Neutrophils isolated from the blood of naïve young and aged mice were stained for neutrophil identification markers (CD45^+^/CD11b^+^/Ly6C^Int^/Ly6G^Hi^), surface chemokine receptors CXCR2 (**A**), CXCR4 (**B**) and the adhesion molecules L-selectin (**C**) and CD44 (**D**). Serum was taken from young (3 month) or aged (22 month) old animals 24 hours following sham or stroke surgery. n=7-12 animals/group. CXCL12 and CXCL2 were measured via multiplex (**E**). Two-way ANOVA was performed, followed by individual T-Tests with Sidak’s correction. (**F**) A schematic figure of the differential roles of CXCL12 and CXCL2 in circulating neutrophil homeostasis. *p≤0.05, **p=<0.01, ***p=<0.001.

One potential reason for augmented reactive species generation in aged blood neutrophils is the delayed clearance of primed neutrophils from the circulation. CXCR2 and CXCR4 control the release of naïve neutrophils from the bone marrow and their return as exhausted apoptotic neutrophils, respectively. Despite no significant age difference in neutrophil CXCR2 expression (Figure 4A), we found that a greater proportion of neutrophils from young animals expressed the bone-marrow homing CXCR4 receptor (Figure 4B, Student T-Test, p=0.0003), and that young CXCR4^+^ neutrophils also had a higher expression of the receptor per cell (Figure 4B, Student T-Test, p=0.0021) than aged neutrophils. In addition, neutrophils from young mice were found to express higher levels of CD44, which promotes neutrophil clearance, on their cell surface compared to those from aged mice (Figure 4C, Student T-Test, p=0.0008). No significant age differences in neutrophil L-selectin were observed (Figure 4D).

We next measured circulating levels of CXCL12 (a bone marrow homing chemokine recognized by CXCR4) and CXCL2, the bone marrow release cytokine recognized by CXCR2 (Figure 4E). A significant interaction effect of age and stroke was seen in CXCL12 levels (Two-way ANOVA, p<0.0001), with aged animals showing higher levels of CXCL12 (p=0.05) under sham conditions, but significantly lower levels of CXCL12 (p=0.006) than young animals after ischemic stroke. No significant effects of age or stroke were seen on levels of CXCL2. A schematic figure illustrating the pattern of CXCL2 and CXCL12, and their cognate receptors CXCR2 and CXCR4, in aged mice is shown (Figure 4F).

### Aged mice display altered circulating levels of neutrophil-associated cytokines after stroke

**Figure 5:**
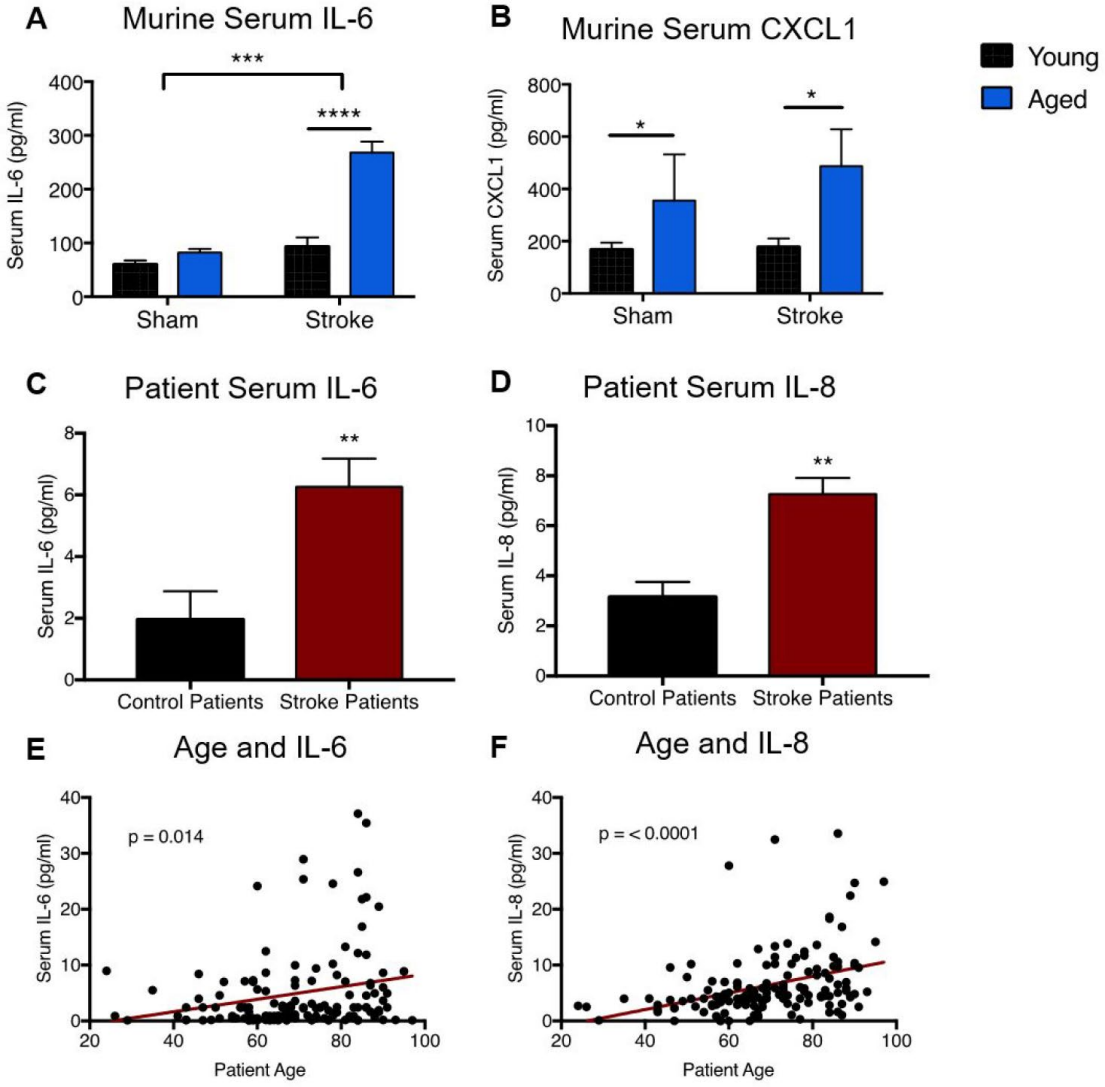
**Aged mice and aged patients display altered circulating levels of neutrophil-associated cytokines after stroke.** Levels of the neutrophil-associated cytokines IL-6 (**A**) and CXCL1 (**B**) in serum from young (3 month) and aged (20-22 month) animals 24 hours after sham or stroke surgery. Data was analyzed by two-way ANOVA, followed by Sidak’s multiple comparisons test. n=3/4 mice per group. Levels of the inflammatory cytokines IL-6 and IL-8 were measured in serum from stroke patients or transient ischemic attack controls 24 hours after stroke onset. Comparison of control (n=17) and stroke (n=143) levels of IL-6 (**C**, Mann-Whitney, p=0.0014) and IL-8 (**D**, Mann-Whitney, p =0.0017). (**B**) Linear correlation between ischemic stroke patient age and levels of IL-6 (**E**, R^2^ = 0.04, p=0.01, n= 143) and IL-8 (**F**, R^2^=0.14, p=<0.0001, n=143) 24 hours after stroke onset. *p≤0.05, **p=<0.01, ***p=<0.001, ****p=<0.0001.

Next, we assessed whether age alters circulating levels of neutrophil-associated cytokines in mice 24 hours after MCAO. Levels of IL-6 (a powerful neutrophil-activating cytokine) and CXCL1 (the primary neutrophil chemoattractant) were measured (Figure 5). By two-way ANOVA, there was a significant stroke effect on serum IL-6 levels (p = <0.0001) in both young and aged animals. A significant age effect (p = <0.0001) and an interaction effect of age/stroke (p=0.0005) was also seen, driven by the augmented increase in IL-6 after stroke in aged animals (p=<0.0001) compared to young animals (Figure 5A). Serum levels of CXCL1, a potent neutrophil chemokine, were found to be significantly elevated in aged animals over young animals, regardless of sham or stroke status (p=0.05, Figure 5B).

### Stroke patient age influences circulating levels of neutrophil-associated cytokines

Given the age-related increases in neutrophil-activating cytokines in mice after stroke, we next examined serum levels of IL-6 and IL-8 (the human homolog of murine CXCL1) in ischemic stroke patients and controls at 24 hours (Supplementary Table 5, Figure 5). Stroke patients were found to have significantly higher levels of serum IL-6 (stroke: 6.26 ± 9.23 pg/ml, control: 1.96 ± 3.77 pg/ml, p=0.0014, Figure 5C) and IL-8 (stroke: 7.27 ± 36.53 pg/ml, control: 3.15 ± 2.47 pg/ml, p=0.0017, Figure 5D) compared to controls (Figure 5C). Within the stroke patient population, we found that increased age was significantly positively associated with higher levels of IL-6 (p=0.015, Figure 5E) and IL-8 (p<0.0001, Figure 5F) in serum 24 hours after stroke. To assess the effects of age independently from other confounding variables (initial stroke severity and patient sex), we performed multivariate multiple regression analysis (Supplementary Table 6). After adjustment, age remained a significant independent predictor of higher IL-8 levels after ischemic stroke (p=0.0008).

### Post-stroke Anti-Ly6G treatment successfully penetrates tissues and depletes circulating neutrophils

The efficacy of anti-Ly6G in depleting neutrophils has been well characterized [21–23]. To confirm that anti-Ly6G produced adequate neutrophil depletion in stroke, sham and stroke mice were treated with 200ug of anti-Ly6G, 500ug of anti-Ly6G or 500ug isotype control antibody at 4, 24 and 48 hours after stroke. Cheek bleeds were obtained at 24 and 48 hours, and cardiac puncture and tissue collection were performed at the 72-hour terminal sacrifice. Flow cytometry utilizing an alternative gating strategy (Supplementary Figure 4) was performed to determine the effects of 200 or 500ug anti-Ly6G on lymphocytes, monocytes and neutrophils in peripheral blood. No significant effects of anti-Ly6G were seen on monocyte or lymphocytes counts (Supplementary Figure 5), Flow cytometry demonstrated that, while 200ug anti-Ly6G was capable of reducing neutrophil proportions in sham animals only 500ug of anti-Ly6G showed significant depletion out to 72 hours in stroke animals (Supplementary Figure 6). We also examined quantitative neutrophil counts using flow cytometry counting beads, confirming the proportional results and demonstrating that neutrophil counts are significantly reduced by anti-Ly6G treatment.

### Anti-Ly6G treatment improves long-term outcomes in aged, but not young, animals

**Figure 6:**
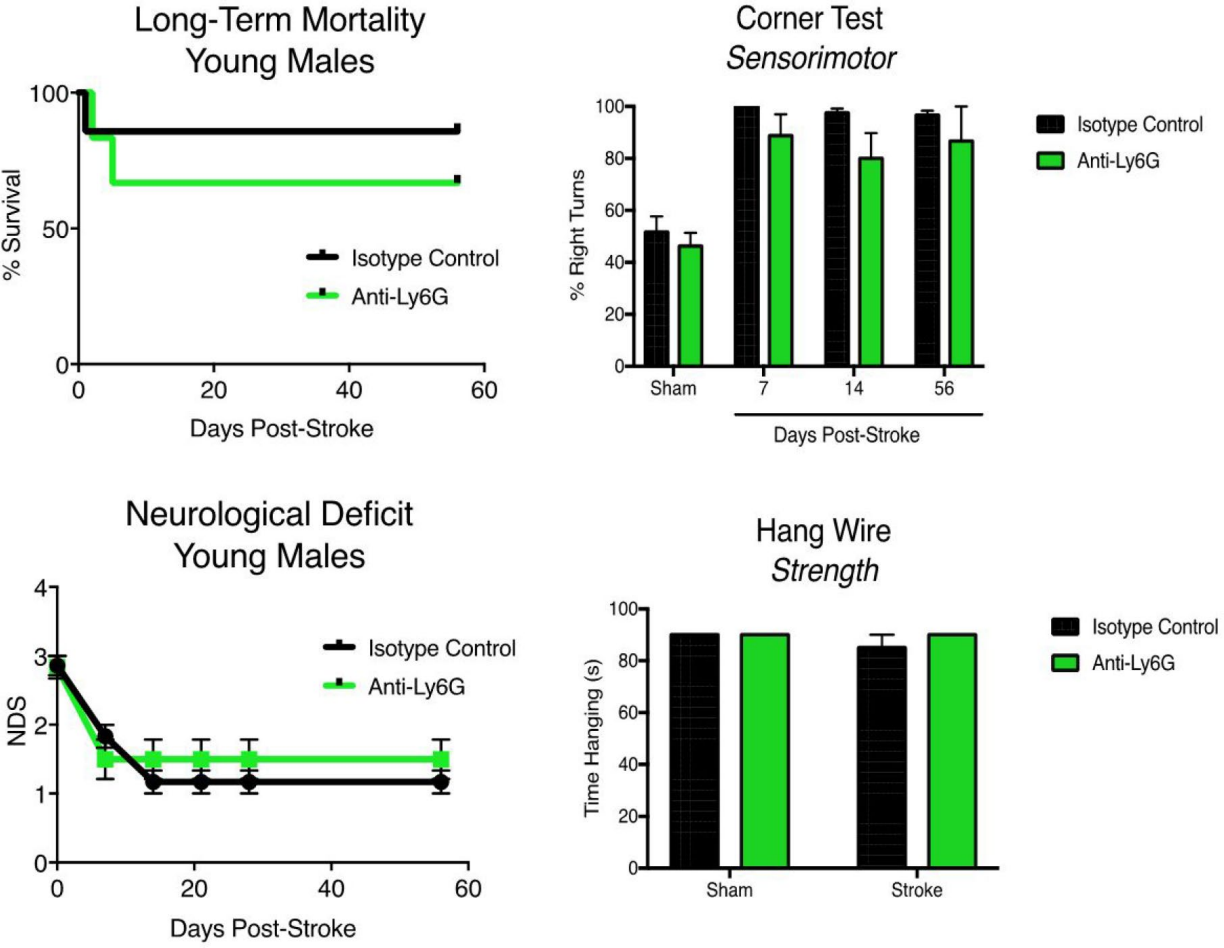
**Anti-Ly6G treatment does not improve long-term mortality, neurological deficits or behavioral testing in young mice after stroke.** Young (3 month) mice were subjected to 60 minute MCAO, and received either 500ug of anti-Ly6G or 500ug of isotype control antibody I.P. at 4, 24 and 48 hours after stroke (n=6-7/group). (**A**) Post stroke mortality was monitored out to 56 days post-stroke. (**B**) Neurological deficits were measured at 7, 14, 21, 28 and 56 days post-stroke. (**C**) Serial corner testing at day 7, 14 and 56 post-stroke in young mice after MCAO. (**D**) Hang-wire strength testing in young mice after MCAO at day 7. N=6-7/group.

**Figure 7:**
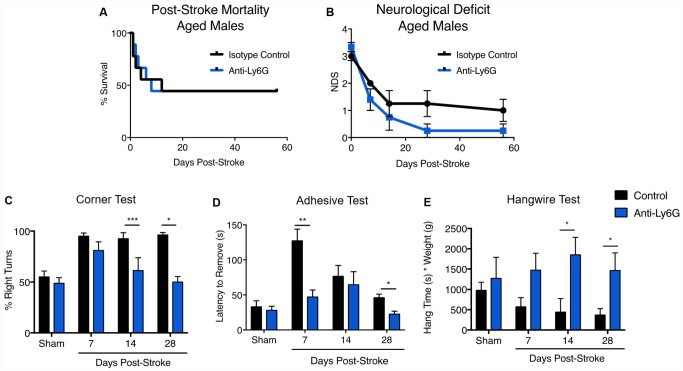
**Anti-Ly6G treatment improves gross neurodeficit recovery after stroke in aged mice. N=9/group.** Mortality (**A**) and neurological deficit score (**B**) were assessed in aged (21-22 month) male mice receiving 500ug of anti-Ly6G or isotype control antibody at 4, 24 and 48 hours after stroke. Behavioral testing of aged mice receiving anti-Ly6G or control antibody after ischemic stroke, including corner test (**C**), adhesive test (**D**) and hangwire test (**E**). *p≤0.05, **p=<0.01, ***p=<0.001, ****p=<0.0001.

Young (3 month) and aged (21-22 months) male mice were subjected to 60 minute MCAO stroke and followed out for two months (Figure 6). No significant effects of anti-Ly6G treatment on long-term mortality (Figure 6A) or neurological deficits (Figure 6B) was seen. In line with the neurodeficit data, young animals showed no benefit of anti-Ly6G treatment on functional outcome after stroke by either corner testing (Figure 6C) or hang-wire testing (Figure 6D). Next, we examined the effects of anti-Ly6G treatment on long-term outcome in aged mice (Figure 7). Despite no differences in long-term mortality (Figure 7A), neutrophil depletion with anti-Ly6G significantly improved gross neurodeficit recovery after ischemic stroke in aged male mice (Figure 7B, p=0.04). Aged animals also demonstrated significant improvement in corner testing at 28 days (Figure 7C, p=0.0002), adhesive removal testing at 7 and 28 days (Figure 7D, 7d p=0.003, 28d p = 0.01) and hang wire testing at 14 and 28 days post-stroke (Figure 7E, 14d p=0.04, 28d p=0.05). Improvements in functional outcome after ischemic stroke after therapeutic delivery can be dependent or independent of gross tissue damage. As stroke size can significantly change functional outcome and all secondary inflammatory sequelae, it is critical to determine whether anti-neutrophil depletion reduces overall direct tissue damage. Cresyl violet staining for cerebral atrophy in brains taken two months after stroke demonstrated that anti-Ly6G treatment did not significantly affect brain atrophy in young or aged mice after ischemic stroke (Supplementary Figure 7). As sex is also known to play a role in inflammation and outcome after ischemic stroke, we also tested the efficacy of anti-Ly6G treatment on long-term stroke outcomes in female mice (Supplementary Figure 8). As in aged males, aged female mice treated with anti-Ly6G showed significantly improved long-term neurological deficits and behavioral recovery.

## DISCUSSION

A wealth of pre-clinical studies indicate that neutrophils can exacerbate brain injury following stroke [24]. Unfortunately, when similar therapeutic strategies have been attempted in clinical trials, no benefit was seen in ischemic stroke patients [5]. This is likely due, in large part, to the fact that many of the targeted molecules are not neutrophil-specific, leading to a high degree of non-specificity. Interestingly, a recent study found that specific genetic deletion of neutrophils (Mcl1 knockout) did not improve neurological deficits after ischemic stroke in mice [25]. However, it is critically important to note that the Mcl1 study, and all previous anti-neutrophil therapies in acute ischemic stroke, were performed only in young animals. As ischemic stroke is largely a disease of aged patients, [2] it is necessary to incorporate aged animals into both mechanistic and therapeutic studies examining neutrophil contributions in ischemic stroke. 

Previous work from our lab found that the aged immune system contributes to poorer stroke outcomes, and that brain-infiltrating neutrophils in aged animals produce higher levels of reactive species than those in young animals [17]. In the present study, we sought to determine how age changes the neutrophil response to ischemic stroke, and whether age influences the direct contribution of neutrophils to stroke pathophysiology.

Our results confirm the strong association between aging and poor outcome after ischemic stroke [2, 17]. In human patients, an association between stroke severity and neutrophilia within 24 hours of stroke was seen. However, in contrast to our predictions, age was not associated with the degree of neutrophilia after stroke in human patients. In agreement with this, our studies in an experimental mouse model found no significant age difference in the (1) magnitude of peripheral neutrophilia or (2) quantitative neutrophil infiltration into the brain at 24 hours post-stroke. This suggests that poor stroke outcomes in aged subjects may be driven by detrimental changes in neutrophil function, rather than overall neutrophil number.

To that end, our subsequent studies focused on the effects of age on neutrophil function. *Ex* *vivo* stimulation found no association between age and reactive species production in neutrophils isolated from bone marrow, but age was found to augment reactive species production in neutrophils taken from the circulation. Together with previous work from our lab showing higher levels of reactive species in aged brain-infiltrating neutrophils, [17] this suggested that detrimental age-related changes in neutrophil reactive species production may be dependent on the local environment.

Neutrophils can exist in three states: quiescent, primed and activated [26]. Under normal conditions, quiescent neutrophils are released from the bone marrow into the circulation, where low-level inflammatory stimuli shift their phenotype from quiescent to “primed”, an intermediate phase characterized by an enhanced inflammatory response (~10x) upon activation with a strong inflammatory stimulus [27, 28]. Neutrophils typically have a very short life span in circulation, with an average half-life of eight hours, [29] facilitating the efficient clearance of primed neutrophils from the bone marrow and limiting unnecessary neutrophil activation [30, 31]. Neutrophil clearance occurs via the upregulation of CXCR4, which recognizes the chemokine CXCL12, and the adhesion molecule CD44 [32, 33]. Flow cytometry and serum chemokine measurements demonstrated that aged animals had lower neutrophil expression of CD44 and the bone marrow return receptor CXCR4, as well as lower serum CXCL12. In addition, flow cytometry revealed neutrophil-skewing of the leukocyte populations in the blood, lung and spleen. Taken together, these results suggest that age mice exhibit enhanced priming of circulating neutrophils, suppression of the normal neutrophil clearance phenotype and an overabundance of neutrophils in the circulation and tissues. Importantly, in conditions of chronic inflammation, neutrophils have been shown to become *inappropriately* primed by the pro-inflammatory systemic inflammatory milieu, [28] potentially resulting in excessive activation and exacerbated pro-inflammatory responses after a major inflammatory insult. Interestingly, Moraga et al. has also reported alterations in neutrophil function in middle-aged animals as compared to young animals in the distal MCAO model of stroke [34].

Our studies demonstrated that age was associated with higher serum levels of IL-6, a pro-inflammatory cytokine that accelerates neutrophil release and migration, in both human stroke patients and our experimental mouse model [35, 36]. This suggests that increased priming of neutrophils in aged animals may be secondary to “inflammaging”, characterized by a chronic low-level increase in circulating pro-inflammatory cytokines like IL-6 in aged individuals [37]. In addition, serum levels of CXCL1/IL-8 were significantly higher in the circulation of aged patients and animals. As this chemokine is a critical promotor of neutrophil chemotaxis, respiratory burst and degranulation, [38, 39] age-associated increases in CXCL1 may contribute to the heightened respiratory burst and altered neutrophil trafficking seen in circulating neutrophils in aged mice.

Finally, using anti-Ly6G, we depleted circulating neutrophils after stroke to assess whether neutrophils play a causative role in poorer outcome in aged animals. No beneficial effects of neutrophil depletion were observed in young animals, but aged animals receiving anti-Ly6G showed significantly improved functional recovery compared to control. Similar benefits were seen in aged female mice receiving neutrophil depletion therapy. Together, these experiments suggest that age increases the detrimental role of neutrophils in ischemic stroke. Future work will explore whether blockade of the potential mediators of enhanced neutrophil pathogenicity (IL-6, CXCL1) identified in these experiments can ameliorate the worse ischemic stroke outcomes seen in aged animals.

This work highlights the importance of using aged animal models, particularly when the target disease occurs largely in an aged population (e.g., cerebral ischemia, atherosclerosis, hypertension). Adapting conclusions made from studies in young animals into treatments for aged humans is likely to decrease the efficacy of clinical translation – and may mask the potential of age-dependent candidate therapies [13, 40, 41]. The implications of age-induced alterations in neutrophil biology and pathogenicity described in our studies are far-reaching, offering potential insight into the role of neutrophils in a variety of age-associated tissue injury and inflammatory processes, including atherosclerosis, cancer and autoimmune disease.

## MATERIALS AND METHODS

### Study approval

The use of de-identified human retrospective data, and the use of human patient bio-samples with written informed consent, was approved by the Institutional Review Board at Hartford Hospital. All animal procedures were performed in accordance with NIH guidelines for the care and use of laboratory animals and approved by the University of Texas Health Science Center at Houston.

### Human subject chart review

Hartford Hospital is a 868-bed community-based teaching hospital certified as a JCO Comprehensive Stroke Center (CSC). Inclusion criteria were defined as ischemic stroke and transient ischemic attack (TIA) patients with known age, initial stroke severity and discharge disposition (n = 3635). TIA was defined as a brief and resolving acute onset focal neurological deficit. TIA patients were chosen as a control group as they have similar characteristics to stroke patients, including co-morbidities and age distribution. Ischemic stroke was defined as acute-onset focal neurological deficits with corresponding evidence of cerebral infarction on radiographic imaging (CT or MRI). Exclusion criteria were defined as ischemic stroke secondary to dissection, iatrogenic stroke or equivocal imaging. Human subject data for the demographic cohort are included in Supplementary Table 1. The primary functional outcome was designated as initial stroke severity, as measured by National Institutes of Health Stroke Score Scale (NIHSS) on admission. Statistical analysis: Linear regression analysis was used to examine the association between patient age and NIHSS admission severity (as a continuous variable). The secondary functional outcome was designated as discharge disposition, as a method of determining mortality (death or discharge to hospice) and morbidity (significant disability requiring significant rehabilitation or long-term care). Discharge disposition was categorized into three groups: Home with or without services (low morbidity), discharge to sub-acute or acute rehab (high morbidity), or death/hospice discharge (death). The relationship between discharge disposition and age was examined via Kruskal-Wallis testing.

### Absolute neutrophil counts

Patients from the demographic data cohort (n=3635) with available lab results were screened for this analysis. Exclusion criteria included active cancer, autoimmune disease, iatrogenic stroke and active immunosuppressive therapy. TIA control (n = 91) and ischemic stroke patients (n = 364) with available absolute neutrophil counts within 24 hours of hospital admission were included in the analysis. Human subject data for the lab value cohort are summarized and included in Supplementary Table 1. Statistical analysis: Differential neutrophil counts were analyzed by two-tailed Mann-Whitney test. The associations of neutrophil counts with age and stroke severity were analyzed by linear regression, followed by multivariate multiple regression to control for potential confounding variables.

### Human serum cytokine measurements

Sample and demographic data collection was conducted at a 868-bed community based teaching hospital certified as a JCO Comprehensive Stroke Center (CSC). Blood was drawn from patients (n = 143) and controls (n=17) at 24 ± 6 hours after symptom onset in tubes containing no anticoagulant. Human subject data for the serum cohort are located in Supplementary Table 5. Blood was allowed to clot at room temperature for two hours before centrifugation and removal of the top layer of serum supernatant. Serum samples were stored at -80°C until use. Levels of IL-6 and IL-8 were measured in patient serum samples (n=160) by Bio Plex Pro Human Cytokine Assay (BioRad).

### Statistical analysis

Differences in two selected serum cytokines (IL-6 and IL-8) between stroke and control patients were analyzed by Mann-Whitney test. Linear regression was then used to examine the univariate relationship between stroke patient age and serum levels of IL-6 and IL-8 at 24 hours. Finally, multivariate multiple regression analysis was performed to assess the independent relationship between stroke patient age and serum IL-6 and IL-8 after adjustment for stroke severity and patient gender as potential confounding variables.

### Animals

Young adult (3-4 mo) and aged (20-22 mo) C57BL/6J male mice were group-housed in a specific pathogen facility on a 12h light/dark cycle. Animals had access to water and chow *ad libitum.* All animal procedures were performed in accordance with NIH guidelines for the care and use of laboratory animals and approved by the University of Texas Health Science Center at Houston.

### Middle cerebral artery occlusion (MCAO)

Animals were randomly assigned to MCAO or control groups. Mice were subjected to focal transient cerebral ischemia by 60 min of reversible MCA occlusion under isofluorane anesthesia by a blinded surgeon [42]. In order to achieve equivalent levels of occlusion, 0.21 mm and 0.23 mm silicone coated sutures (Coating Length 2.5mm, Doccol) were used to occlude the MCA in young and aged mice, respectively. Rectal temperatures were maintained at approximately 37° C during surgical ischemia with an automated temperature control feedback system. For verification of adequate ischemia, cerebral blood flow (CBF) was measured via Laser Doppler flow measurement (Moor Instruments). Animals were sacrificed 24 hours post-stroke (for acute infarct) [20] or 2 months post-stroke (for long-term atrophy quantification).

### Neurological deficit scores and behavioral testing

Neurological scores were recorded in mice at the time of reperfusion, and then once daily for seven days post-MCAO surgery. Neurological scoring was performed by an observer blinded to treatment group. In long-term outcome studies, neurological scoring was repeated at 7, 14, 21, 28 and 54 days. The scoring system is as follows: 0, no deficit; 1, forelimb weakness and turning of the torso to the ipsilateral side when held by tail; 2, circling towards the affected side; 3, unable to bear weight on affected side; and 4, no spontaneous locomotor activity or barrel rolling, as previously described. [43].

### Corner test

The corner test is a measure of integrated sensorimotor function, involving both sensory stimulation and motor response. The mouse was placed between two plastic pieces with dimensions of 12 x 24 inches. The two boards were placed at an angle of 30° and gradually moved closer to the mouse, encouraging the subject to move into the created corner. This resulted in stimulation of the vibrissae on both sides of the mouse’s face, causing the mouse to rear up and turn back towards the open end. Each mouse was tested in 20 separate trials, and the percentage of right turns was calculated.

### Hanging wire test

In this test, mice utilize all four limbs to support their body weight on a wire-cage elevated 36 inches above a cage containing soft bedding. The time until the mouse fell was recorded, with an upper limit of 90 seconds. Mice unable to hang were excluded from the study. For trials involving aged mice, the average hang time was corrected for weight, to adjust for the weight variability within the cohort.

### Adhesive removal test

In this test, a small round adhesive circle measuring 1cm was applied to the left forepaw of a mouse. The mouse was then placed alone in a clean cage. The time to remove the adhesive sticker was measured, with an upper limit of 5 minutes.

### Euthanasia and tissue collection

Mice were anesthetized using 2,2,2- Tribromoethanol (Sigma-Aldrich, St. Louis, MO). Arterial blood was harvested via intracardiac puncture into a 1ml syringe pre-coated with 1,000U heparin sulfate, and serum and plasma were collected for cytokine analysis (described below).

### Acute infarct quantification

Aged and young animals were sacrificed 24 hours post-stroke. Following sacrifice, brains were removed and chilled for 4 minutes at -80°C to harden the tissue before slicing. Brains were then sliced into 2mm coronal sections, and stained with 1.5% 2,3,5-triphentyltetrazolium (TTC, Sigma-Aldrich) for 20 minutes at 37*C. Sections were then washed with PBS before fixation overnight in 4% paraformaldehyde at 4°C before digital imaging (Sigma Scan Pro5) and infarct volume measurement (ImageJ) by a blinded investigator [44, 45].

### Cresyl violet atrophy quantification

In cresyl violet cohorts, transcardial perfusion with cold, heparinized PBS was followed by 4% paraformaldehyde. Brains were collected, fixed in 4% cold paraformaldehyde for 24 hours, then placed into a 30% sucrose solution to dehydrate. Following dehydration, brains were frozen and sliced into 30-um slices on a freezing microtome, with every eighth slice mounted and stained with cresyl violet for the evaluation of brain atrophy. Digital images were taken and infarct volumes were measured via computer software (Sigma Scan Pro5).

### Flow cytometry

For flow cytometric studies, animals were transcardially perfused with 60ml of sterile, cold PBS. The organs of interest (blood leukocytes, brain, lungs, femoral bone marrow and spleens) were subsequently collected and rinsed with cold, sterile PBS before processing. Cells were stained, and neutrophils identified as CD45^+^/CD11b^+^/Ly6C^Int^/Ly6G^+^ cells. Cells were further analyzed for intracellular ROS production, or surface phenotypic marker staining. Arterial blood was subjected to three rounds of 10 minute RBC lysis on ice using Tris-ammonium Chloride (Stem Cell Technologies, Cambridge, MA) at 9:1, 4:1 and 4:1 stringencies. The remaining white blood cells were washed twice with 500ul of cold PBS, then placed in 1ml of supplemented RPMI (Life Technologies) containing L-glutamate, 2.5% HEPES Buffer, 1X Pen-Strep, 5% heat-inactivated FBS until staining. Spleens and bone marrow were removed, mechanically disrupted and filtered through a 70um filter screen, followed by 1 round of RBC lysis with Tris-ammonium chloride. Brains were collected and the olfactory bulbs, brainstem and cerebellum were removed. The cerebrum was divided into two hemispheres, and the ipsilateral (stroke) hemisphere was prepared for flow cytometry. Hemispheres were mechanically processed with a razor blade before enzymatic digestion in collagenase/dispase (Roche Diagnostics) and DNAse (Roche Diagnostics) at 37°C for 1 hour. Following incubation, the cells were filtered through a 70um filter screen, followed by leukocyte enrichment via density centrifugation (70%/30% Percoll, GE Lifesciences). Cells were washed twice with ice-cold PBS, followed by a 30 minute RT incubation with an amine-reactive viability stain (Ghost Dye 510, Tonbo Biosciences). Cells were spun down and re-suspended in FACS Buffer (PBS + 2% FBS) containing Fc Receptor Blocker CD16/32, 1:100 for 10 minutes at room temperature.

### Neutrophil identification flow cytometry panel

Following this blocking step, cells were re-suspended in the following cocktail of fluorescently conjugated antibodies: CD45-vf450 (Tonbo Biosciences), CD11b APC-Cy7 (Tonbo Biosciences), Ly6G-PeCy (Tonbo Biosciences) and Ly6C-PeCF594 (BD Biosciences) and incubated for 30 minutes at room temperature. After staining, cells were washed twice and re-suspended in 300ul FACS buffer for analysis on a Cytoflex S Flow Cytometer (Beckmann Coulter). Data files were analyzed using FlowJo (TreeStar). The representative gating strategy used is given in Supplementary Figure 2. Positive and negative populations were defined using fluorescence minus one (FMO) controls.

### Neutrophil isolation

Mice were anesthetized and transcardially perfused with 60ml ice-cold PBS prior to cervical dislocation. All long bones of the forelimbs and hindlimbs were dissected out, cleaned of muscle and fat and washed in 70% ethanol. Bones were then crushed in a small mortar and pestle containing 5ml of Hanks Balanced Salt Solution (HBSS) without calcium and magnesium, supplemented with 2% heat-inactivated fetal bovine serum, in order to remove the bone marrow. The resulting bone marrow homogenate was filtered through a 40um cell strainer into a 50ml falcon tube, then washed twice with HBSS + 2% FBS. The bone marrow cells were then re-suspended in 1ml room temperature HBSS and layered onto a gradient made of Histopaque-1.077 (3ml) and Histopaque-1.119 (Sigma). Cells were centrifuged at 500g for 30 minutes at room temperature with the brake off, mononuclear cells at the HBSS/1.077 interface were removed and discarded, and neutrophils were collected at the 1.077/1.119 interface. Cells were then washed twice with HBSS + 2% FBS and re-suspended in 500ul HBSS before negative magnetic isolation via the Stem Cell Mouse Neutrophil Isolation Kit (Stem Cell Technologies).

### Cytokine analysis

Isolated neutrophils were incubated with 0, 50 or 200 nM PMA at 37°C for 45 minutes. After stimulation, the supernatant was collected and frozen down at -80°C until use. Samples were thawed and chemokine levels were measured in plasma using the Bio-Plex Pro Mouse Chemokine Assay (Bio-Rad), on a Bio Plex 200 Luminex System. Cytokine and chemokines with detectable supernatant levels were then analyzed by multiple T-testing, with correction for multiple comparisons, in order to determine the effects of PMA stimulation on neutrophil chemokine production.

### In vivo ROS assay

Single-cell brain and bone marrow suspensions were washed twice in 1X sterile, cold PBS, followed by viability staining with Ghost Dye 510 (Tonbo Biosciences) for 30 minutes in the dark at room temperature. Cells were centrifuged at 500g for 5 minutes at 4°C, the supernatant was discarded and the pelleted cells were re-suspended in 100ul of 1:100 FC Receptor Block (Tonbo Biosciences) for 10 minutes at room temperature. Cells were then stained with following surface antibodies: CD45-vf450, CD11b-APC-Cy7, Ly6G-Pe-Cy7, Ly6C-APC (Tonbo Biosciences) for 30 minutes at room temperature, protected from light. Following surface staining, samples were incubated with dihydrorhodamine 1,2,3 (DHR) and 200 nM PMA for 45 minutes at 37°C according to the manufacturer’s instructions (Neutrophil Monocyte Respiratory Burst Assay Kit, Cayman Chemical), then washed and analyzed immediately for rhodamine fluorescence on a Beckmann Coulter Cytoflex S Flow Cytometer. Data was analyzed using FlowJo (TreeStar).

### Ex vivo ROS assay

Neutrophils isolated as described above were then counted and diluted to a concentration of 1x10^6 cells/ml. Cells were stained with surface markers mentioned above (CD45, CD11b, Ly6G, Ly6C). Following surface staining, dihydrorhodamine 1,2,3 dye (DHR), PMA and incubation buffer were used according to manufacturer’s instructions (Neutrophil Monocyte Respiratory Burst Assay Kit, Cayman Chemical). 100ul of cells were taken and incubated with DHR and various concentrations of PMA (0, 25, 50, 100, 200, 400 nM PMA) for 45 minutes at 37°C. Flow cytometry was used to identify neutrophils, and measure median fluorescence intensity of the DHR signaling per cell.

### Neutrophil surface marker phenotyping flow cytometry panel

Blood was drawn from naïve young (3 month) and aged (22 month) mice via cardiac puncture and prepared as above. In addition to neutrophil quantification markers described above and in Supplementary Figure 1, cells were also stained with an antibody cocktail containing four neutrophil surface markers: CD62L-FITC (Biolegend), CXCR4 PerCP-Cy5.5 (Biolegend), CXCR2 PE (Biolegend) and CD44 PE-Dazzle (Biolegend) and incubated for 30 minutes at RT. After staining, cells were washed twice and re-suspended in 300ul FACS buffer for analysis on a Cytoflex S Flow Cytometer (Beckmann Coulter). Data was analyzed as described above.

### Serum and plasma cytokine measurements

Blood was drawn from anesthetized young and aged mice 24 hours after sham or stroke surgery via cardiac puncture. Blood for plasma preparation was drawn with a 1ml syringe/18g needle pre-coated with heparin (1000U), and collected into a tube containing 50ul of diluted (200U) heparin. The tube was gently inverted several times at room temperature before centrifugation at 4°C for 20 minutes at 15,000 RPM. After centrifugation, the top layer of plasma was removed and stored at -80°C until testing. Chemokine levels were measured in plasma using the Bio-Plex Pro Mouse Chemokine Assay (Bio-Rad), on a Bio Plex 200 Luminex System. Two neutrophil chemokines were selected for further analysis (CXCL2 and CXCL12) by two-way ANOVA. In order to best match the serum cytokine data from our human cohort, we elected to analyze levels of circulating inflammatory cytokines in serum. Blood for serum preparation was drawn into a clean 1ml syringe/18g needle into a tube without anti-coagulant, and allowed to clot at room temperature for two hours before centrifugation and removal of the serum supernatant. Serum was stored at -80°C until analysis. The levels of IL-6, CXCL1 (the two cytokines identified to change with age in stroke patients) and G-CSF (an important neutrophil growth factor) were assessed using the Bio-Plex Pro Mouse Cytokine Assay (Bio-Rad), on a Bio-Plex 200 Luminex System. Data was analyzed by two-way ANOVA.

### Drug administration

Mice were randomized to receive either 500ug anti-Ly6G monoclonal antibody (Clone 1A8, BioXCell) or isotype control antibody (Clone 2A3, BioXCell) intraperitoneally (I.P.) at 4, 24 and 48 hours after sham or MCAO stroke. For the dose determination experiment, an additional group of mice receiving 200ug of anti-Ly6G antibody was added. Investigators were blinded to treatment. Confirmation of depletion was confirmed by flow cytometry.

### Neutrophil depletion confirmation

Flow cytometry was performed in order to determine the efficacy of Ly6G tissue penetration and circulating neutrophil depletion. Sham and stroke mice received 500ug anti-Ly6G or isotype control antibody at 4, 24 and 48 hours after sham or ischemic stroke surgery. Cheek bleeds were performed on all mice at 24 hours and 48 hours, and blood was prepared for flow cytometry. Following red blood cell lysis, cells were stained as outlined in Chapter 3, and CountBrite (Thermofisher) counting beads were added to samples before analysis in order to determine absolute neutrophil numbers. In the treatment group that received anti-Ly6G antibody for neutrophil depletion, the surface epitope of Ly6G remains highly bound by the depletion antibody, masking the presence of neutrophils by traditional gating. Therefore, we developed an alternative gating strategy that did not rely on Ly6G for neutrophil identification (CD45^+^/CD11b^+^/SSC^High^/ Ly6C^Intermediate^). Our alternate gating strategy is presented in Supplementary Figure 4. At 72 hours, mice were sacrificed, and blood was obtained by cardiac puncture. To assess tissue penetration, spleen and bone marrow were also harvested. All three tissues were prepared for flow cytometry, and our alternative gating strategy was applied.

### Statistics

### Human retrospective data

Linear regression analysis was used to examine the association between patient age and NIHSS admission severity (as a continuous variable). The secondary functional outcome was designated as discharge disposition, as a method of determining mortality (death or discharge to hospice) and morbidity (significant disability requiring significant rehabilitation or long-term care). Discharge disposition was categorized into three groups: Home with or without services (low morbidity), discharge to sub-acute or acute rehab (high morbidity), or death/hospice discharge (death). The relationship between discharge disposition and age was examined via Kruskal-Wallis testing. Human neutrophil counts: Differential neutrophil counts were analyzed by two-tailed Mann-Whitney test. The associations of neutrophil counts with age and stroke severity were analyzed by linear regression, followed by multivariate multiple regression to control for potential confounding variables. Human cytokine levels: Differences in two selected serum cytokines (IL-6 and IL-8) between stroke and control patients were analyzed by Mann-Whitney test. Linear regression was then used to examine the univariate relationship between stroke patient age and serum levels of IL-6 and IL-8 at 24 hours. Finally, multivariate multiple regression analysis to assess the independent relationship between stroke patient age and serum IL-6 and IL-8 after adjustment for stroke severity and patient gender as potential confounding variables. Animal study statistics were analyzed by two-tailed T-test, ANOVA or Kruskal-Walls testing, where appropriate (GraphPad, Prism).

## Supplementary Material

Supplementary Figures

Supplementary Tables
