# Supplementary material for: Aging exacerbates neutrophil pathogenicity in ischemic stroke
Source: Aging (Albany NY). 2020 Jan 12;12(1):436–61. doi: 10.18632/aging.102632 (PMC6977697; doi:10.18632/aging.102632)
Supplement: Supplementary Figures [file aging-12-102632-s002..pdf]

SUPPLEMENTARY FIGURES

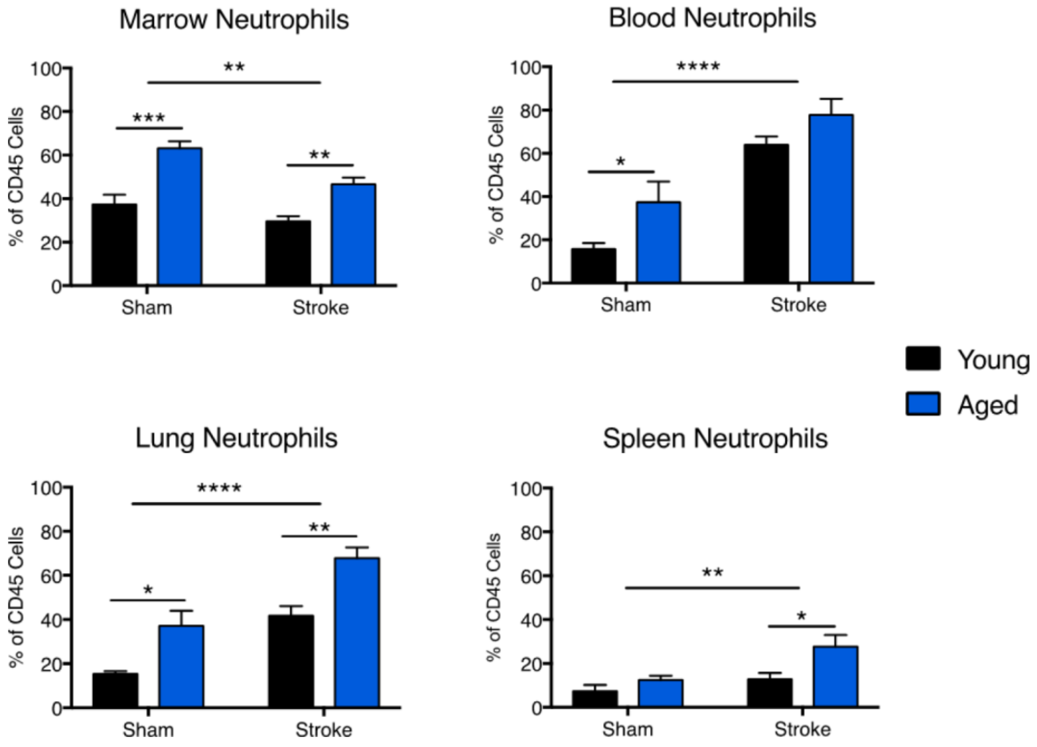

Supplementary Figure 1. Age and stroke significantly alter neutrophil proportions across multiple organs. Neutrophils in the bone marrow, blood, lungs and spleen of young (3 month) or aged (22 month) mice were identified by flow cytometry 24 hours after stroke or sham surgery. Neutrophils were identified as $CD45^{+}/CD11b^{+}/Ly6C^{Int}/Ly6G^{Hi}$ cells. *=p<0.05, **p<0.01, ***=p<0.001, ****p<0.0001. n=3-6/grp.

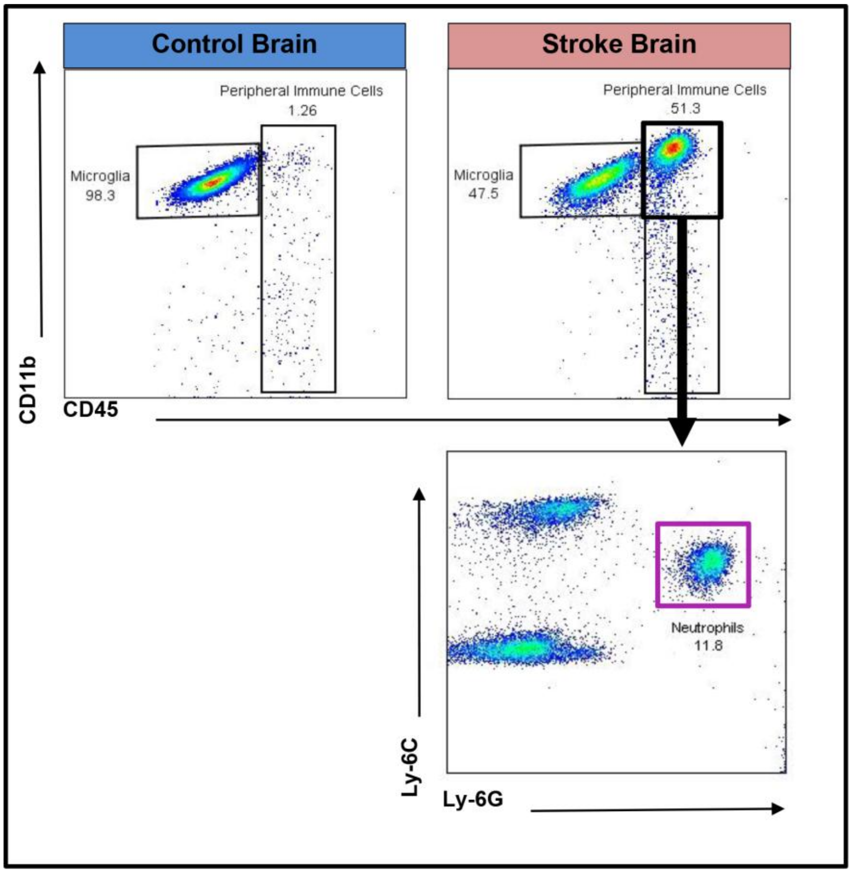

Supplementary Figure 2. Gating strategy for neutrophil identification (brain). Following gating for singlets and viability, immune cells are identified by their expression of the pan-immune cell marker CD45 and their expression of the myeloid lineage marker CD11b. Microglia, the resident immune cells of the brain, are identified as $CD11b^{+}/CD45^{Intermediate}$ cells. Peripheral immune cells are identified as $CD45^{High}$ cells, with peripheral myeloid cells identified as $CD11b^{+}$/ $CD45^{High}$. Out of these peripheral myeloid cells, neutrophils are then defined as $Ly6C^{Intermediate}/Ly6G^{High}$.

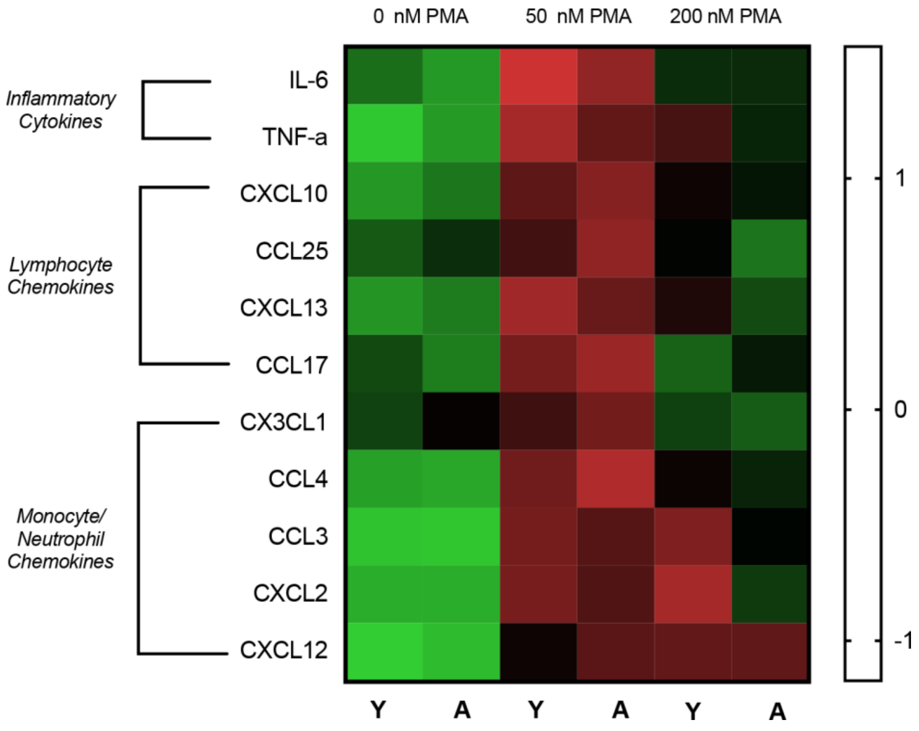

Supplementary Figure 3. Heat map of differentially expressed cytokines and chemokines in young and aged cultured neutrophils. Neutrophils were isolated from murine bone marrow and stimulated for 1 hour with 0, 50 or 200 nM PMA. Chemokine and cytokine production was measured in the culture supernatant by multiplex ELISA.

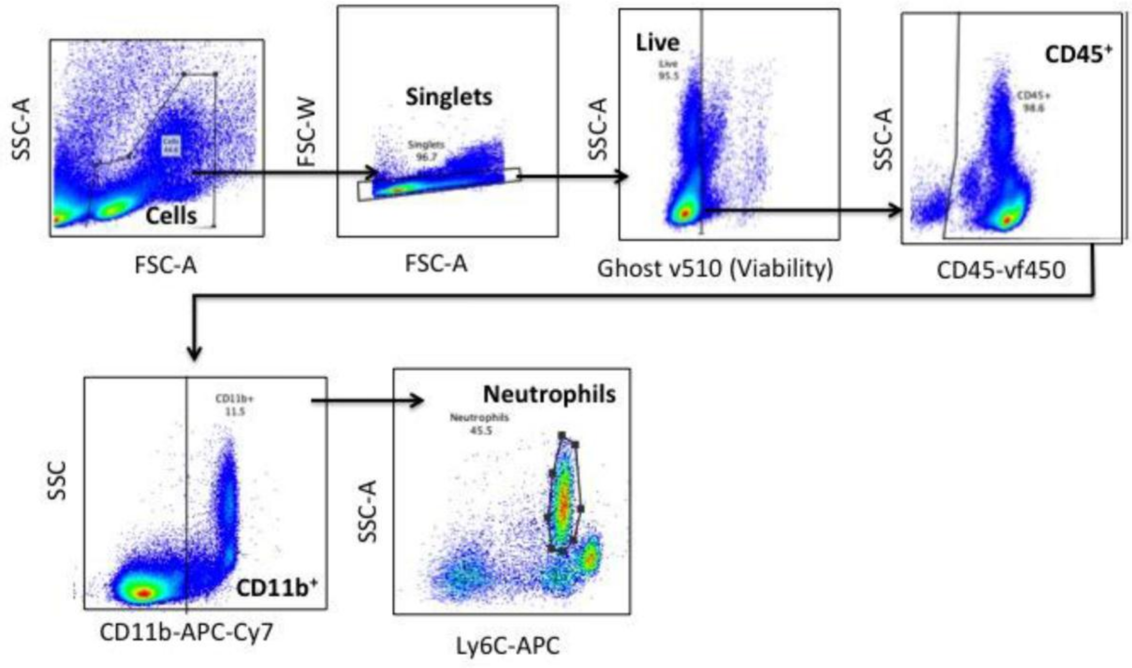

Supplementary Figure 4. Alternative gating strategy for anti-Ly6G neutrophil depletion confirmation (blood). In order to identify neutrophils in the neutrophil depletion cohort, we utilized an alternative gating strategy. Live, single cells were selected for CD45 and CD11b expression to classify myeloid cells, followed by identification of neutrophils as Ly6C^{Int}and SSC^{High} cells, with inflammatory monocytes representing the Ly6C^{High} and SSC^{Int} population

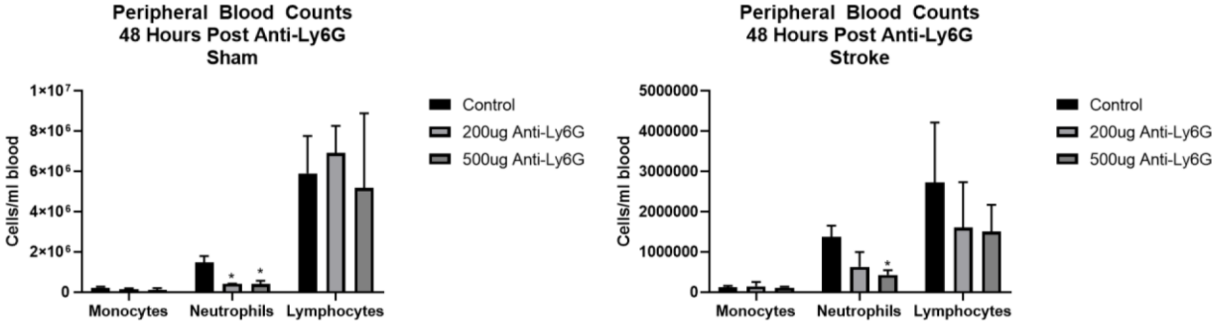

Supplementary Figure 5. Monocyte, lymphocyte and neutrophil counts from peripheral blood in sham and stroke animals receiving control IgG, 200ug anti-Ly6G or 500ug anti-Ly6G. Data was analyzed by 2-tailed student T-test. n=3/4 per group.

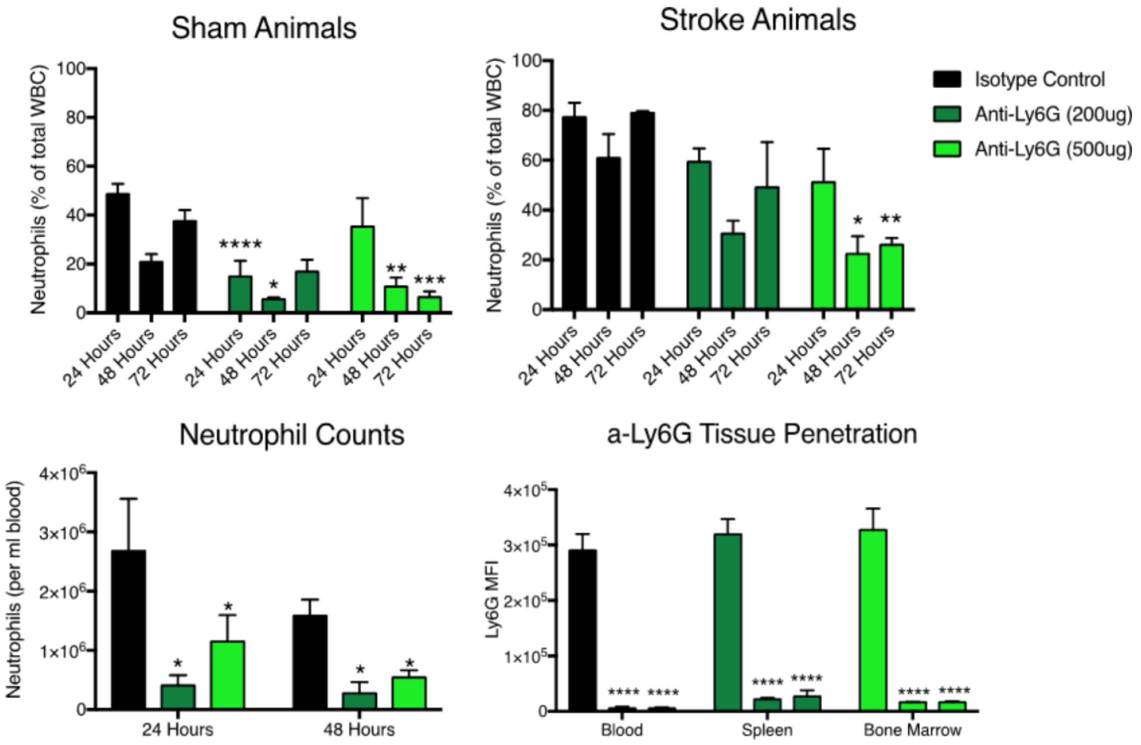

Supplementary Figure 6. Neutrophil depletion efficacy of anti-Ly6G. Young mice were subjected to sham or stroke MCAO (60 minutes), then treated with either isotype control antibody, 200ug of anti-Ly6G or 500ug of anti-Ly6G antibody I.P. at 4 hours, 24 hours and 48 hours after stroke. Data was analyzed by two-way ANOVA, followed by Tukey's multiple comparisons testing. (**A**) Reduction of neutrophil proportions in treated sham animals. (**B**) Reduction of neutrophil proportions in treated stroke animals. (**C**) Absolute neutrophil counts in animals treated with isotype control, anti-Ly6G (200ug) or Anti-Ly6G (500ug). (**D**) Ly6G median fluorescence intensity in blood, spleen and bone marrow. n=3-4/grp.

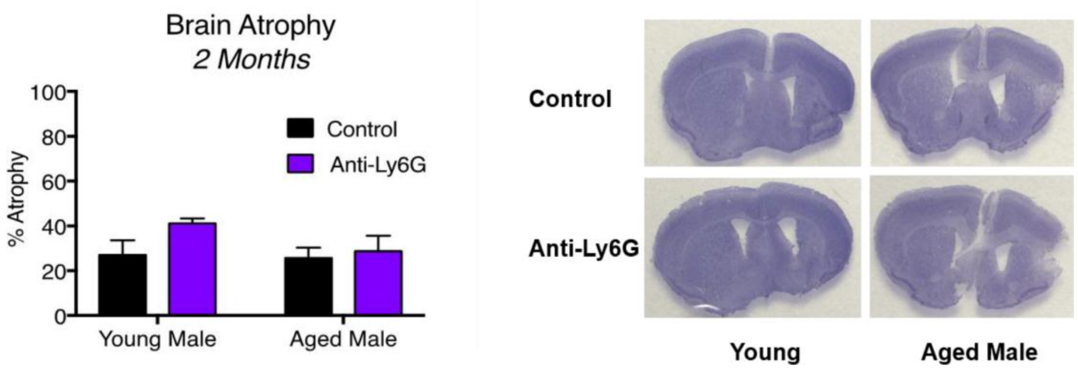

Supplementary Figure 7. Neutrophil depletion does not alter long-term brain atrophy after ischemic stroke. Young (3 month) and aged (22 month) mice received 500ug or anti-Ly6G at 4h, 24h and 72h after stroke. Two months after stroke onset, mice were sacrificed and tissue atrophy was quantified by cresyl violet histological staining.

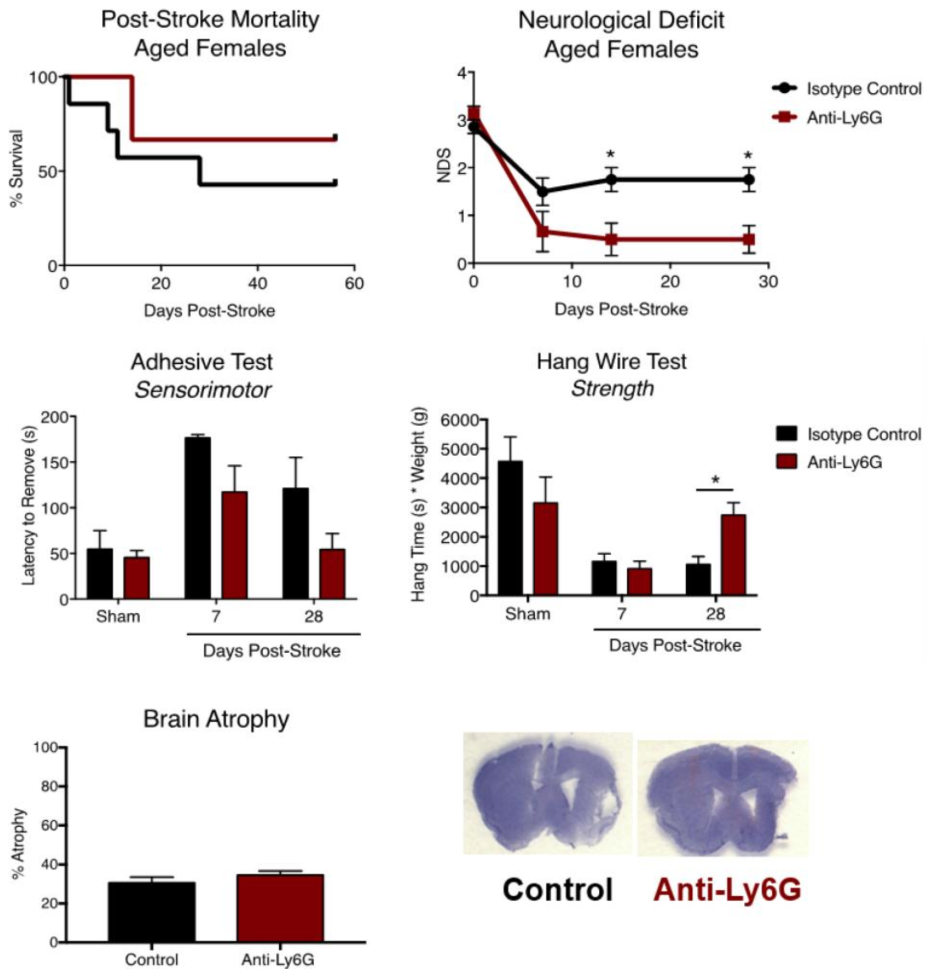

Supplementary Figure 8. Anti-Ly6G treatment improves outcomes in aged female animals after stroke. Aged female mice received treatment with either 500ug anti-Ly6G antibody or isotype control I.P. at 4, 24 and 48 hours after stroke. (**A**) Post-stroke mortality of aged females. (**B**) Neurological deficit measurements in aged female animals. (**C**) Adhesive testing of aged female animals at 7 days and 28 days post-stroke. (**D**) Hang-wire strength testing of aged female animals at 7 days and 28 days post-stroke. (**E**) Atrophy analysis of CV stained slices taken 2 months post-stroke. (**F**) Representative CV images for atrophy quantification. N=3-6/group.
