# Supplementary material for: Aging exacerbates neutrophil pathogenicity in ischemic stroke
Source: Aging (Albany NY). 2020 Jan 12;12(1):436–61. doi: 10.18632/aging.102632 (PMC6977697; doi:10.18632/aging.102632)
Supplement: Supplementary Tables [file aging-12-102632-s001..pdf]

SUPPLEMENTARY TABLES

Supplementary Table 1. Patient characteristics for stroke outcome and neutrophil count studies.

Cohorts	Demographic cohort	Absolute neutrophil count
**Age** (Mean ± SD)	69.3 ± 15.6 yr	75.7 ± 9.9 yr
**Sex** (Female, %)	50.5 %	50.3 %
**N**	3635	508

The average age, sex composition and n is given for the demographic outcome and absolute neutrophil count cohorts.* SD = Standard Deviation; yr = year.

Supplementary Table 2. Multivariate regression analysis of the relationship between patient age, sex, stroke severity (as determined by patient age at admission) and neutrophil count in ischemic stroke patients 24 hours after ischemic stroke (n=508).

ANOVA

	d.f.	*SS*	*MS*	*F*	*p-level*
Regression	3.	280,197,188.74448	93,399,062.91483	11.3797	3.1204E-7
Residual	504.	4,136,589,476.413	8,207,518.80241		
Total	507.	4,416,786,665.15748			

	Coefficient	*Standard Error*	*LCL*	*UCL*	*t Stat*	*p-level*	*H0 (5%)*
**Intercept**	5,774.53342	641.54271	4,514.106	7,034.96084	9.00101	0.	*rejected*
**Age**	-0.92512	8.91175	-18.43387	16.58364	-0.10381	0.91736	*accepted*
**Sex**	-234.68639	261.57773	-748.60345	279.23068	-0.8972	0.37004	*accepted*
**NIH Admit**	101.44329	17.91046	66.25493	136.63165	5.66391	2.48935E-8	*rejected*
T (5%)	1.96468						

LCL - Lower value of a reliable interval (LCL)

UCL - Upper value of a reliable interval (UCL)

Supplementary Table 3. Ischemic stroke model parameters in young and aged mice.

Age	Weight (g)	CBF reduction (%)	Temperature (C)
**Young** (3 months)	26.24 ± 1.11	89.54 ± 2.70	36.60 ± 0.46
**Aged** (20-22 months)	34.68 ± 1.93	89.86 ± 1.76	36.82 ± 0.55
**Significance**	p=0.0002	n.s.	n.s.

Young and aged mice were subjected to right MCAO stroke surgery. Weight (mean ± SD), cerebral blood flow (CBF) reduction (% of baseline, mean ± SD) and temperature during stroke (mean ± SD) are given.

Supplementary Table 4. Bone marrow neutrophil production of inflammatory chemokines and cytokines with or without PMA stimulation for 45 minutes (n=3-4/group).

Analyte (pg/ml)	0 nM PMA	50 nM PMA	q value	FC
CXCL2	214.3 ± 2.31	248.60 ± 64.76	<0.0001	32.92
CCL3	104.3 ± 1.27	122.30 ± 43.18	<0.0001	26.75
CXCL12	373.5 ± 20.07	323.40 ± 112.11	0.0001	10.66
CCL4	38.41 ± 3.54	69.87 ± 23.80	0.0001	3.44
IL-6	2.97 ± 0.42	5.59 ± 1.16	<0.0001	1.59
TNF-a	14.65 ± 2.60	18.90 ± 2.72	<0.0001	1.39
CCL17	2.89 ± 0.76	4.93 ± 0.83	0.0002	0.97
CXCL13	199.80 ± 31.42	256.30 ± 32.92	0.002	0.62
CCL24	131.60 ± 19.62	101.90 ± 33.79	n.s.	0.57
CCL5	15.14 ± 3.60	12.78 ± 5.96	n.s.	0.55
CXCL10	83.23 ± 11.10	101.30 ± 9.58	0.0001	0.51
CCL25	174.40 ± 36.75	231.00 ± 43.93	0.03	0.36
CCL20	6.73 ± 1.26	6.85 ± 1.19	n.s.	0.28
CX3CL1	19.86 ± 4.51	26.15 ± 2.10	0.04	0.26
CCL1	2.03 ± 0.50	2.67 ± 0.79	n.s.	0.25
CCL27	306.40 ± 77.50	413.30 ± 45.75	n.s.	0.18
CCL7	1.32 ± 0.49	1.83 ± 0.28	n.s.	0.05
IFN-y	4.90 ± 2.20	6.02 ± 0.99	n.s.	0.01
IL-10	38.60 ± 17.62	63.20 ± 14.52	n.s.	-0.01
CXCL11	33.46 ± 17.88	41.88 ± 9.40	n.s.	-0.08
IL-16	86.37 ± 25.70	77.87 ± 45.03	n.s.	-0.21
IL-1B	30.04 ± 17.16	28.39 ± 9.24	n.s.	-0.3

Supplementary Table 5. Patient characteristics for serum cytokine (Il-6 and IL-8) measurement studies.

	TIA control patients	Ischemic stroke patients
**Age** (Mean ± SD)	64.5 ± 11.13 yr	69.3 ± 14.7 yr
**Sex** (Female, %)	25.0%	47.0%
**N**	17	143

The average age, sex composition and n is given for the patients included in the serum cytokine analysis in Chapter 4. * SD = Standard Deviation; yr = year.

Supplementary Table 6. Multivariate regression of serum cytokine levels (IL-6 and IL-8) in ischemic stroke patients.

	Serum IL-6			Serum IL-8		
	Coefficient	*SE*	*p-level*	*Coefficient*	*SE*	*p-level*
**Intercept**	-2.5	3.06	0.41	-3.95	2.33	0.09
**Sex**	1.77	1.22	0.14	0.74	0.93	0.42
**Age**	0.01	0.04	0.83	0.11	0.03	0.0008
**Severity**	0.47	0.07	1.09E-8	0.15	0.05	0.009

Multivariate regression was performed to assess the influence of age on serum IL-6 and IL-8 24 hours after stroke onset on ischemic stroke patients, controlling for the effects of age and stroke severity.
